# Dual Impact: Coup–Contrecoup Brain Injury in a Young Cyclist—A Case Report

**DOI:** 10.1155/cris/9963242

**Published:** 2026-07-08

**Authors:** Abhishek Kumar, Shashikant Prasad, Majid Anwer, Anurag Kumar, Anil Kumar

**Affiliations:** ^1^ Department of Trauma Surgery and Critical Care, All India Institute of Medical Sciences, Patna, Bihar, India, aiims.edu

**Keywords:** case report, coup–contrecoup brain injury, decompressive craniectomy, Glasgow coma scale, traumatic brain injury

## Abstract

**Introduction:**

Coup–contrecoup brain injury occurs when trauma produces focal damage both at the site of impact (coup) and at the contralateral side (contrecoup) due to intracranial pressure (ICP) dynamics and rotational forces. These injuries are frequently seen in high‐velocity accidents and often demand staged neurosurgical intervention.

**Case Presentation:**

We report a case of a 14‐year‐old boy presenting with altered sensorium following a road traffic accident. Initial CT revealed a large left frontal extradural hematoma (EDH) with mass effect and contralateral right frontotemporoparietal contusion. He underwent emergency left frontal craniotomy and evacuation of ~150 mL of EDH. Despite initial improvement, the patient developed neurological deterioration with anisocoria on postoperative day three. Repeat CT revealed worsening contralateral contusion with subdural hematoma (SDH) and midline shift. A right frontotemporoparietal decompressive craniectomy with lax duraplasty was performed, resulting in resolution of mass effect. The patient showed progressive neurological recovery, was decannulated, discharged on postoperative day 18, and later underwent elective cranioplasty with good functional outcome.

**Conclusion:**

Coup–contrecoup injuries are mechanistically linked to rotational acceleration, ICP gradients, and skull deformation, rather than simple rebound forces. Frontal and temporal lobes are most commonly involved. Clinical predictors of outcome include Glasgow coma scale (GCS), pupillary reactivity, presence of SDH, and systemic secondary insults. These injuries are dynamic, with contralateral lesion progression often necessitating vigilant monitoring and timely intervention. Management requires repeat neuroimaging, control of ICP, and staged surgical procedures when indicated.

## 1. Introduction

Coup–contrecoup brain injury refers to focal cerebral damage occurring both at the site of impact (coup) and at the contralateral side (contrecoup), resulting from complex interactions of linear and rotational acceleration, intracranial pressure (ICP) gradients, and skull deformation [1–4]. Contemporary biomechanical evidence suggests that rotational forces and shear strain, rather than simple rebound mechanisms, are primarily responsible for contralateral lesion formation [5–7].

These injuries are commonly seen in high‐velocity trauma and are associated with significant morbidity. The frontal and temporal lobes are particularly susceptible due to their proximity to irregular skull base contours, which amplify stress during rapid deceleration[2, 3]. In pediatric patients, coup–contrecoup injuries are especially important due to their dynamic nature and the potential for delayed progression of contralateral lesions, which may not be fully evident on initial imaging [8].

This case is unique for several reasons. It demonstrates a classic yet clinically challenging coup–contrecoup pattern in a pediatric patient, with initial presentation dominated by a surgically significant extradural hematoma (EDH) and subsequent delayed progression of the contralateral contusion requiring staged neurosurgical intervention. The case highlights the dynamic evolution of traumatic brain injury (TBI), the importance of vigilant monitoring and repeat neuroimaging, and the need for timely decision‐making regarding secondary decompressive surgery. Furthermore, the favorable neurological outcome despite the severe initial injury underscores the potential benefits of an aggressive, staged management approach in carefully selected patients.

## 2. Case Presentation

**Figure 1:**
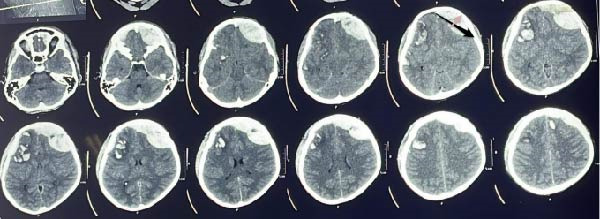
Left frontal extradural hematoma (EDH), arrow with mass effect, right frontotemporoparietal contusion (A = black arrow, B = gray arrow, C = number of slices containing hematoma × slice thickness).

A 14‐year‐old boy was brought to the Emergency Department (ED) of AIIMS Patna on 13/12/24 at 5: 30 PM; 2 h postinjury after being struck on the side by a speeding two‐wheeler while cycling. He was evaluated based on the advanced trauma life support (ATLS) guidelines. On the primary survey, his airway was threatened due to a low level of consciousness. The cervical spine was immobilized. He was placed on mechanical ventilation. His pulse was 88 bpm and BP was 95/54 mm Hg. His Glasgow coma scale (GCS) was E1VtM4, pupils 4 mm, reactive; cervical spine was immobilized. There was no external bleeding, and mild hypothermia was prevented. On secondary survey, he had bilateral periorbital ecchymosis (“raccoon eyes”). There was a laceration (2 cm× 0.2 cm) over the left temporal region. The abdomen, chest, and pelvis were found to be unremarkable. A noncontrast computed tomography (NCCT) of the head showed a left frontal EDH measuring ~5.5 cm in maximal thickness with a craniocaudal extent of ~ 5.5 cm and anteroposterior extent of ~ 10 cm, consistent with a high‐volume lesion. Using the ABC/2 method, the estimated volume of the left frontal EDH was ~150 mL. It was associated with effacement of adjacent cortical sulci and a midline shift of ~4 mm toward the right side. In addition, a contralateral right frontotemporoparietal hemorrhagic contusion was noted, characterized by patchy hyperdense areas with surrounding hypodense edema, without a significant mass effect or midline shift at initial presentation (Figure 1).

**Figure 2:**
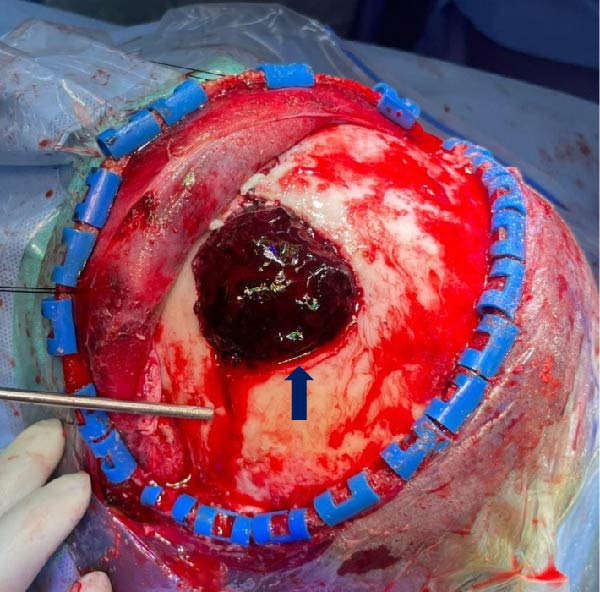
Intraoperative image showing evacuation of extradural hematoma (arrow).

**Figure 3:**
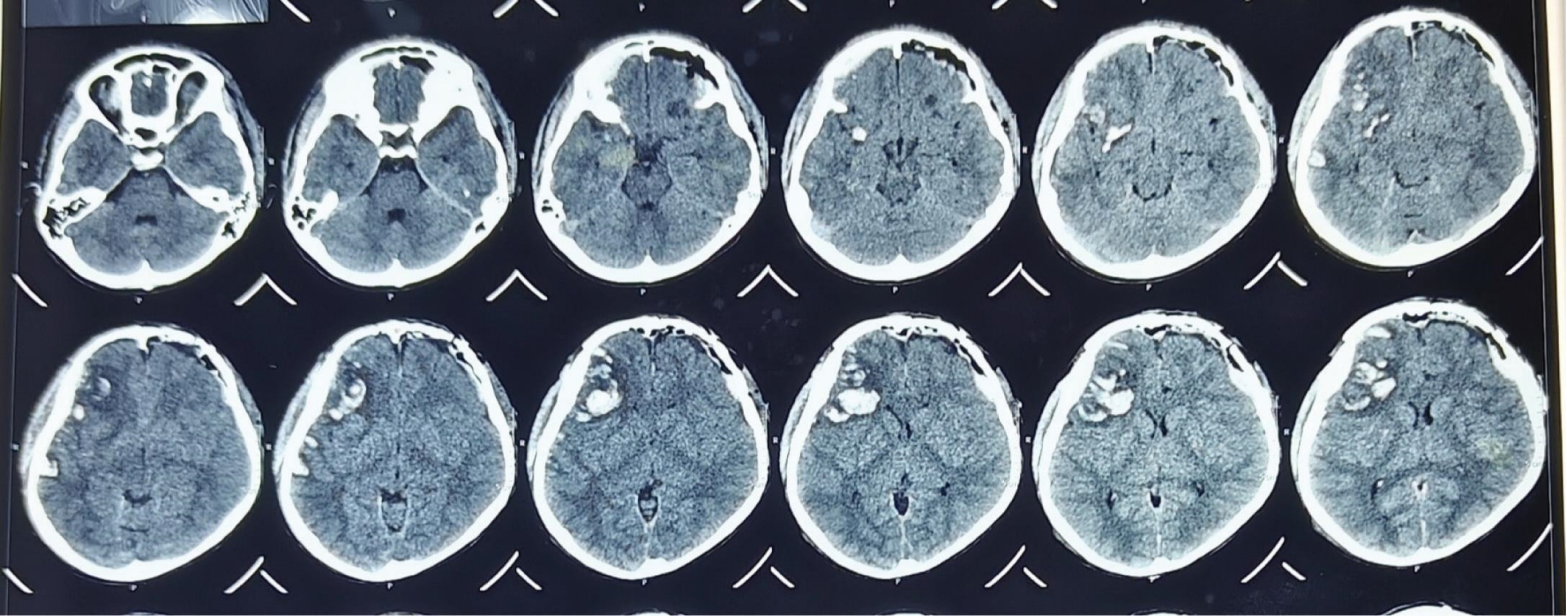
Resolved EDH and right‐sided frontotemporoparietal contusion with mild mass effect.

Chest and pelvic X‐ray were found to be unremarkable. NCCT: the cervical spine was unremarkable, and the cervical collar was removed. A baseline laboratory parameters revealed hemoglobin of 8.7 g/dL (12–16 g/dL), total leukocyte count of 21.36 × 10^3^/µL (4–11 × 10^3^/µL), platelet count of 105 × 10^3^/µL (150–400 × 10^3^/µL), prothrombin time of 21.7 s (11–15 s), and an international normalized ratio (INR) of 1.64. In view of the planned emergency neurosurgical intervention, coagulopathy was addressed preoperatively with appropriate blood product support to reduce the risk of intraoperative bleeding. The patient’s anemia was also taken into consideration in the context of TBI and transfused blood products as per the protocol. The patient was diagnosed as a case of left frontal large EDH with mass effect and was planned for urgent craniotomy. An emergency left frontal craniotomy with the evacuation of 150 mL of EDH was performed (Figure 2). Haemostasis was secured, dural hitch was taken, bone flap was replaced and fixed with plates and screws, along with a subgaleal drain placed. He was kept intubated and shifted to the ICU. He was put on mechanical ventilation. In the postoperative course, immediate postoperative GCS was E2VTM5. A post op NCCT head on 14/12/24 was done, which showed resolution of left EDH and right‐sided frontotemporal contusion with mild mass effect (Figure 3).

**Figure 4:**
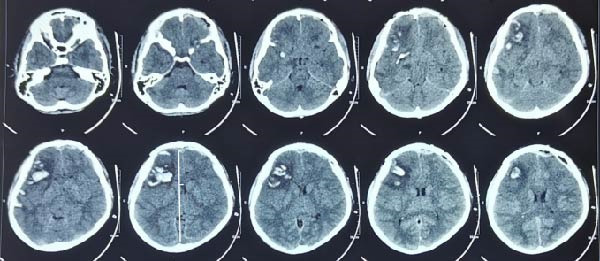
Right frontotemporal contusion and midline shift of 6mm (white line and white arrow).

He was placed on strict GCS and pupil charting. Invasive ICP monitoring was not performed. Clinical management was guided by neurological examination and radiological findings, including trends in the GCS, pupillary reactivity, and evidence of mass effect and midline shift on serial neuroimaging. The initiation of hyperosmolar therapy with mannitol and hypertonic saline was based on suspected intracranial hypertension, as indicated by the depressed sensorium and evolving radiological features. He was started on hyperosmolar therapy with 20% mannitol 100 mL IV tid and 3% NaCl @10 mL/hr via infusion. The patient remained hemodynamically stable afterwards throughout the hospital stay. Tracheostomy was performed on POD 2 due to poor neurological recovery and the need for prolonged mechanical ventilation. On 16/12/24, his GCS dropped to E2VtM4 with anisocoria; CT revealed a worsening right frontotemporoparietal contusion with a midline shift of 6 mm and subdural hematoma (SDH) with edema (Figure 4).

**Figure 5:**
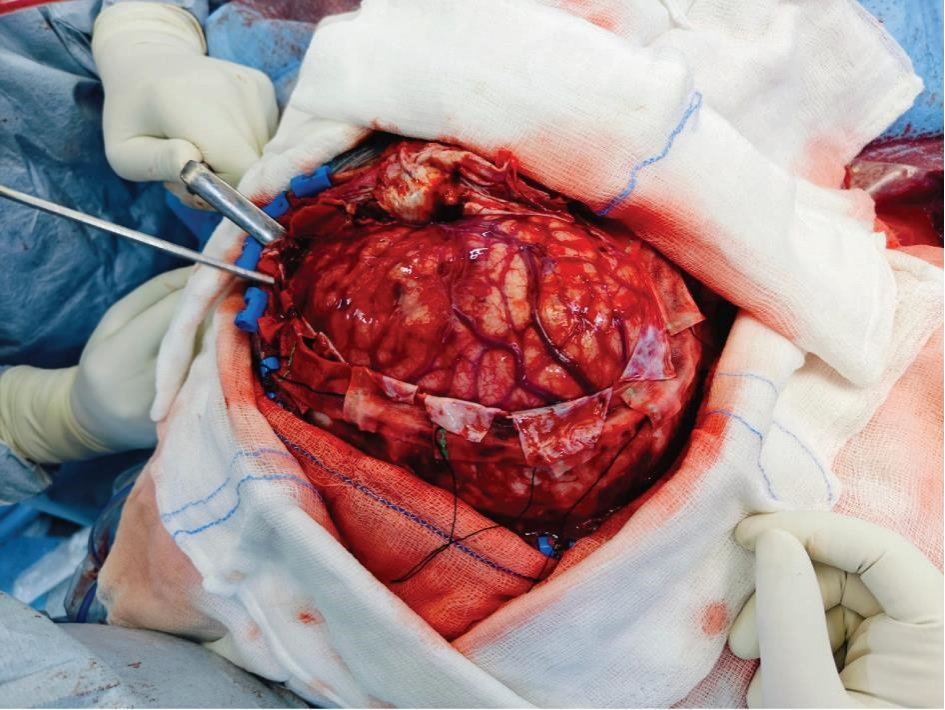
Intraoperative image of right decompressive craniectomy.

**Figure 6:**
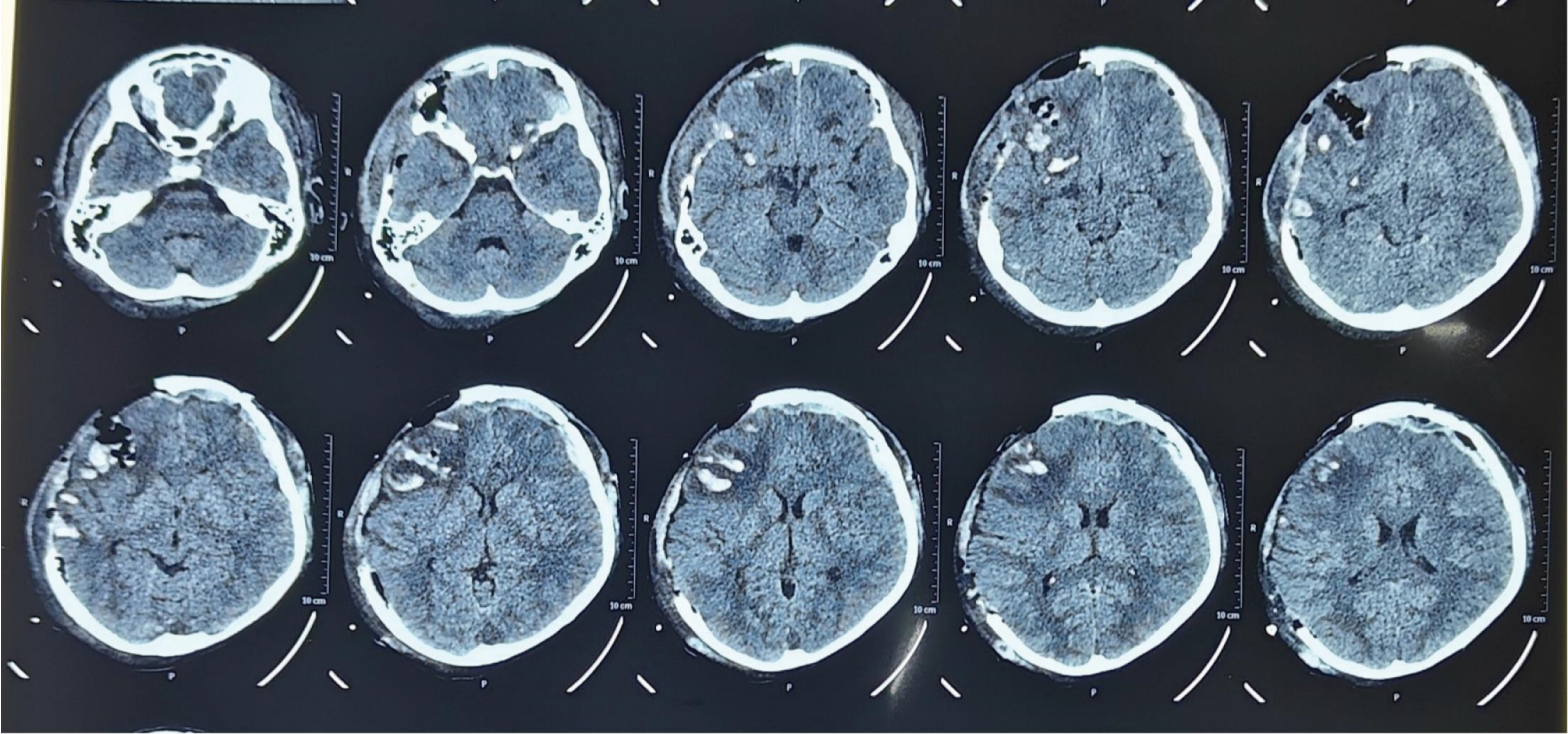
Post OP NCCT showing right decompressive craniectomy and resolution of midline shift.

The patient was again immediately shifted to the OR. Right frontotemporoparietal decompressive craniectomy with lax duraplasty was performed (Figure 5). The brain appeared tense with a contusion and thin SDH; however, no active bleed was noted. A repeat NCCT head on 17/12/24 showed resolution of the midline shift (Figure 6).

The patient was shifted to the ICU in an intubated state. On 18 December, his GCS improved to E4VtM6. He was weaned off ventilatory support, and tracheostomy was later decannulated. He was later shifted to the ward and discharged on 31 December. He came for follow‐up at 1, 2, and 3 months. He had a neurological status of GCS E4V5M6, reactive pupils. There was no sensory or motor deficit in any of the limbs. He later underwent elective titanium mesh cranioplasty with good recovery (Table 1). The functional outcome was classified as Glasgow outcome scale (GOS) score of 5 (good recovery) at 3 months, indicating resumption of normal life without significant disability.

**Table 1:** Timeline of clinical course, neurological status, imaging, and interventions.

Time point/POD	GCS score	Pupillary status	Key clinical events	Imaging findings	Interventions
Admission (day 0)	E1VtM4	4 mm, reactive bilateral	Altered sensorium following RTA; intubated and ventilated	NCCT: large left frontal EDH (~3 cm thickness, ~4 mm MLS), right frontotemporoparietal contusion, skull fractures	Emergency left frontal craniotomy and EDH evacuation (~150 mL)
POD 1	E2VtM5	Equal, reactive	Hemodynamically stable, under ICU monitoring	NCCT: EDH evacuated; residual right‐sided contusion with mild edema, no significant MLS	Continued ventilation, hyperosmolar therapy
POD 2	E2VtM5	Equal, reactive	Poor neurological recovery; prolonged ventilation anticipated	—	Tracheostomy performed
POD 3 (deterioration)	E2VtM4	Anisocoria	Acute neurological decline	NCCT: progression of right contusion with SDH and ~ 6 mm MLS	Emergency right frontotemporoparietal decompressive craniectomy with duraplasty
POD 4	—	—	Immediate postoperative stabilization	NCCT: resolution of midline shift	ICU care continued
POD 5	E4VtM6	Equal, reactive	Neurological improvement	—	Gradual weaning from ventilator
POD 11	E4VtM6	Equal, reactive	Stable neurological status	—	Shifted to ward
POD 18 (discharge)	E4V5M6	Equal, reactive	Clinically stable, no deficits	—	Discharged
Follow‐up (1–3 months)	E4V5M6	Equal, reactive	No neurological deficits	—	Elective cranioplasty performed

## 3. Discussion

Coup–contrecoup brain injury represents a well‐recognized but still debated pattern of focal TBI, characterized by contusions at the site of impact (coup) and at the opposite pole of the brain (contrecoup). The phenomenon was first described more than a century ago [8] and has since been re‐examined by neuropathologists, biomechanical engineers, and clinicians seeking to clarify its mechanisms and prognostic implications. Coup–contrecoup injuries in pediatric populations are relatively uncommon in younger children due to increased skull elasticity and open sutures, with the incidence increasing with age as skull rigidity develops. This addition helps place our adolescent case within the broader clinical and biomechanical context of pediatric TBI.

Traditional explanations emphasized direct brain–skull impact and rebound forces. Courville [1] observed that skull deformation and ICP transients contributed significantly to lesion localization. Gurdjian and Gurdjian [3] and Drew and Drew [4] challenged the oversimplified “brain sloshing” model, instead highlighting the role of complex ICP gradients and tissue strain. Experimental work by Morrison et al. [8] and Sabet et al. [5] provided biomechanical support for rotational acceleration and shear deformation as primary mechanisms, particularly in generating contralateral contusions. Recent computational models have reinforced these insights. Ramzanpour et al. [6] demonstrated through finite‐element simulations that contralateral stresses often exceed those at the site of impact. Toma et al. [7] similarly showed that a “knock‐out punch” produces contralateral strain concentrations, underscoring the importance of angular acceleration. Collectively, these findings suggest that coup–contrecoup injuries are best understood as deformation‐driven phenomena modulated by rotational forces, skull geometry, and boundary effects rather than simple translational rebound.

Clinically, coup–contrecoup injuries most commonly affect the frontal and temporal lobes, regions vulnerable due to their proximity to bony ridges. In an Indian series, Bhateja et al. [9] identified the GCS score, pupillary reactivity, hypotension, cisternal effacement, and acute SDH as key predictors of outcome, with mortality and morbidity largely determined by secondary systemic insults and mass effect rather than the coup–contrecoup pattern per se.

Srivastava et al. [10], in their recent study on occipitofrontal contrecoup injuries, highlighted the high incidence of lesion progression and neurological deterioration. They emphasized the importance of vigilant monitoring, serial imaging, and timely decompressive interventions. These findings reinforce the dynamic nature of coup–contrecoup contusions, where hemorrhagic progression of contusion (HPC) and intracranial hypertension frequently dictate the outcome.

Management strategies parallel those of severe TBI but with heightened attention to lesion evolution. Repeat CT scans within 6–24 h are often warranted given the risk of HPC [10, 11]. ICP monitoring and aggressive control of secondary insults remain central. Early neurosurgical intervention should be considered for patients with expanding contusions, significant mass effects, or associated SDHs [9, 10].

Our institutional protocol emphasizes frequent neurological assessment (GCS and pupillary monitoring), early identification of clinical deterioration, and repeat imaging triggered by neurological changes. While routine scheduled repeat CT is not universally performed, this case highlights that a lower threshold for early repeat imaging (<72 h) may be beneficial in high‐risk patients, consistent with recommendations from prior studies. In our patient, neurological deterioration on postoperative day 3, characterized by a drop in GCS and anisocoria, prompted immediate repeat neuroimaging, which demonstrated significant progression of the contralateral contusion with a mass effect and midline shift. The progression of the contralateral contusion in this patient is most consistent with HPC. Initial impact causes microvascular disruption within the contused brain parenchyma; over time, these injured vessels can bleed further, leading to the expansion of the lesion. This process is driven by secondary injury mechanisms, including evolving cerebral edema, loss of autoregulation, and local coagulopathy. Compared with adults, the pediatric skull is thinner and more elastic, allowing greater transmission and distribution of impact forces rather than localized dissipation. This increased deformability may facilitate wider propagation of strain and shear forces across the brain, predisposing to contralateral injury and potentially contributing to the delayed evolution of contusions. The patient was taken for urgent decompressive craniectomy without delay, in line with established neurosurgical principles.

An additional clinical implication is the need for systematic contralateral surveillance. Biomechanical and clinical evidence suggest that contralateral lesions may be radiologically occult at presentation but evolve subsequently, particularly in high‐energy deceleration or rotational injuries [4–7]. Rehabilitation planning must also consider the potential for diffuse axonal injury and cognitive deficits that frequently coexist with coup–contrecoup mechanisms.

The evolution of our understanding of coup–contrecoup injury reflects the integration of neuropathology, biomechanics, and clinical outcomes research. From early descriptive pathology [1, 8] to modern finite‐element modeling [5–7], it is clear that rotational acceleration and intracranial boundary conditions are central to pathogenesis. Clinically, the coup–contrecoup designation does not independently determine the prognosis but serves as a red flag for contralateral lesion development and the need for serial imaging. Prognosis is ultimately dictated by injury severity, systemic factors, and timeliness of intervention [9–11].

## 4. Conclusion

Coup–contrecoup injuries frequently demonstrate dynamic evolution, with delayed progression of contralateral lesions even after initial surgical management. This case highlights the importance of vigilant neurological monitoring, early consideration of repeat imaging in high‐risk patients, and timely surgical intervention when deterioration occurs. Favorable recovery, including successful delayed cranioplasty, can be achieved with appropriate staged management. However, these observations are based on a single case and should be interpreted as practical clinical insights rather than generalized recommendations.

## Author Contributions

Abhishek Kumar and Shashikant Prasad were involved in the treatment, data collection, and writing of the case report. Majid Anwer and Anurag Kumar combined all the data, figures, and discussion to write the final article. Anil Kumar was involved in the critical analysis of the data as well as the manuscript.

## Funding

The authors declare no external/internal funding for the project.

## Ethics Statement

As per institutional policy (AIIMS Patna), formal ethics committee approval is not required for single‐case reports. Written informed consent for publication was obtained from the patient’s legal guardian as the child is a minor.

## Conflicts of Interest

The authors declare no conflicts of interest.

## Patient Perspective

The patient and his family were thankful for the prompt treatment and clear communication provided by the healthcare team during this critical illness. They were satisfied with the favorable recovery and gradual return to normal daily activities following the staged surgical interventions.

## Data Availability

All the data supporting the findings of this study are included within the article. Additional anonymized clinical data may be made available by the corresponding author upon reasonable request in accordance with institutional policies and patient confidentiality requirements.
